# Translational Prospects and Challenges in Human Induced Pluripotent Stem Cell Research in Drug Discovery

**DOI:** 10.3390/cells5040046

**Published:** 2016-12-21

**Authors:** Masaki Hosoya, Katherine Czysz

**Affiliations:** Integrated Technology Research Laboratories, Research Division, Takeda Pharmaceutical Company Limited, 26-1, Muraoka-Higashi 2-chome, Fujisawa, Kanagawa 251-8555, Japan

**Keywords:** iPS cells, drug discovery, translational research, biomarker

## Abstract

Despite continuous efforts to improve the process of drug discovery and development, achieving success at the clinical stage remains challenging because of a persistent translational gap between the preclinical and clinical settings. Under these circumstances, the discovery of human induced pluripotent stem (iPS) cells has brought new hope to the drug discovery field because they enable scientists to humanize a variety of pharmacological and toxicological models in vitro. The availability of human iPS cell-derived cells, particularly as an alternative for difficult-to-access tissues and organs, is increasing steadily; however, their use in the field of translational medicine remains challenging. Biomarkers are an essential part of the translational effort to shift new discoveries from bench to bedside as they provide a measurable indicator with which to evaluate pharmacological and toxicological effects in both the preclinical and clinical settings. In general, during the preclinical stage of the drug development process, in vitro models that are established to recapitulate human diseases are validated by using a set of biomarkers; however, their translatability to a clinical setting remains problematic. This review provides an overview of current strategies for human iPS cell-based drug discovery from the perspective of translational research, and discusses the importance of early consideration of clinically relevant biomarkers.

## 1. Introduction

**Figure 1:**
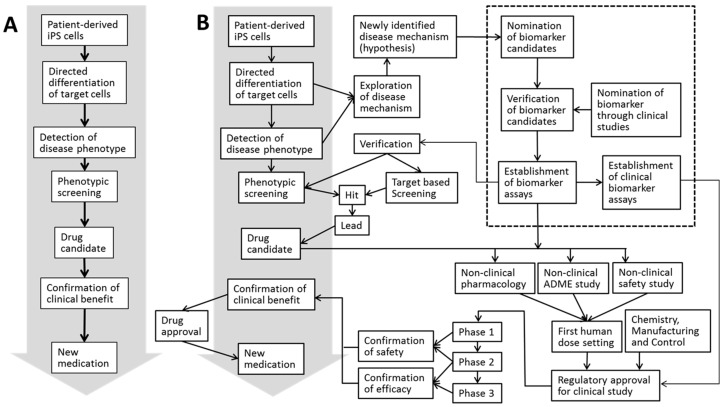
Schematic illustration of the development process from patient-derived induced pluripotent stem (iPS) cells to a new medication. Outline (**A**) and addition of the processes necessary from both the scientific and regulatory perspectives. (**B**) Biomarker strategy is highlighted by the dotted box.

Stem cells are on a fast track to becoming an indispensable source of cells in the fields of both regenerative medicine and drug discovery [1]. To date, these cells have been used as an in vitro tool in a variety of applications, including compound screening, pharmacology and toxicology testing. Pluripotent stem cells (PSCs), such as embryonic stem (ES) cells, embryonic carcinoma cells and embryonic germ cells, can be distinguished from other types of stem cells on the basis of their concomitant capacities to self-renew and to differentiate into almost any cell type but placenta [2]. Among these, ES cells have the most significant effect in the field of drug discovery, not only as a tool for in vitro studies but also as a means to establish genetically modified animals that can be utilized in both in vivo pharmacology and disease characterization experiments [3]. However, the ethical concerns surrounding human ES cells [4] have hampered their continuous use, thus shifting attention toward induced pluripotent stem (iPS) cells. In 2006, iPS cells were derived by introducing a specific set of pluripotency-associated genes, called “Yamanaka’s factors,” into adult mouse fibroblasts [5]. The discovery of these reprogramming factors was a revolutionary breakthrough because these adult mammalian cells were the first to be efficiently reprogrammed to the pluripotent state [6]. The subsequent establishment of human iPS cells [7,8,9,10] has gained a great deal of attention in the field of drug discovery and development, particularly at the earliest stage of the process, because these cells can be used to humanize a variety of pharmacological and toxicological models in vitro. iPS cells have ushered in a new era of translational medicine because they can be used to generate patient-derived pluripotent stem cell lines that can recapitulate not only disease phenotypes but also the process of disease development [11]. Importantly, even though Retro- and Lenti-viral vectors have been the first methods of choice to reprogram somatic cells to pluripotency while still offering one of the highest reprogramming efficiencies, utilizing non-integrating, zero-footprint methods—such as those based on mRNA—miRNA, episomal vectors, and Sendai virus have brought iPS cell (iPSC)-derived cells closer to the clinical application stage [12,13,14]. These improved methods for iPSC reprogramming prevent the risk of vectors randomly integrating into the host’s genome, with no noticeable differences in the frequency and the type of karyotypic change observed [15]. Moreover, even though iPSC epigenetic memory is one of the issues preventing full realization of clinical aspects of iPSC research, a recent study by Kyttala et al. [16] suggests that the donor variability surpasses source-specific iPSC differences. The availability of normal or diseased human iPS cell-derived cells, particularly as alternatives for difficult-to-access tissues and organs, is expanding, and the “disease in a dish” approach is highly anticipated to contribute to the discovery and development of new medicines [17]. Figure 1A shows a schematic illustration of the process in which patient-derived iPS cells can be applied to generate new medications. The drug discovery process comprises multiple tightly regulated stages throughout which promising compounds are discovered and developed, and iPS cells can be applied at numerous stages of such standardized drug discovery workflow (Figure 1B). In short, the process of drug discovery and development typically starts with screening for hit molecules followed by the identification and selection of a handful of lead molecules. The subsequent optimization and rate-limiting steps aim to improve the efficacy, safety, clinical benefits, and industrial manufacturing process before any of the remaining lead compounds can enter the clinical trial stage [18]. Bringing a new chemical entity (NCE) onto the market requires an enormous investment of both time and money [19], and the late stages of clinical trials consume the majority of the investment.

Despite continuous efforts to improve the efficiency of each stage of the drug discovery process, the success at the clinical stage remains challenging. Approximately 90% of investigational drugs entering Phase 1 fail as a result of the translational gap between the preclinical and clinical settings [20]. A root-cause analysis of suspended programs has revealed that a major cause of drug failure during the most costly Phase 3 trials is efficacy (54%), and the main causes of failure during the subsequent new drug application/biologics license application stage are efficacy (48%) and safety (31%) [21]. There are various reasons for such a high drug attrition rate in clinical trials; however, most of these reasons are based on the inability to detect compound toxicity during nonclinical safety assessment of drug. The current status and future potential applications of human iPS cell-derived models in the fields of disease understanding and drug discovery have been extensively discussed in recent years [22,23,24]. Translational research (TR) has emerged as a new paradigm for the drug discovery process because it continues to change the landscape of the field by bridging clinical and basic research [25,26]. The need to improve animal model systems has long been debated [27]; however, the humanization of drug discovery tools by applying human iPS cells is expected to rapidly improve translational value [23]. Along with in vitro and in vivo models, biomarkers are an essential part of TR [28,29], including the application of iPS cells. Many reviews have described the promise of iPS cells in the drug discovery field; however, many of them have focused on isolated stages of this process. It would be useful to both scientists and patients to elucidate the integrated potential of iPS cells and biomarkers across the entire process of drug discovery and development and to highlight the areas in need of further improvement.

## 2. Biomarkers in Translational Research (TR)

In a broad sense, a biomarker is any type of a measurable biochemical, morphological, physiological or behavioral characteristic of an organism. The definition of a biomarker in biomedical research is still under discussion, but a broad consensus has been established [30] and is often aligned with goals of the stakeholders [31]. Protein (expression, localization, and modification) and mRNA (expression) are the most popular biomarkers used in in vitro studies with iPS cells, to evaluate the quality and method development of directed differentiations. In this sense, the first biomarkers applied to iPS cells were Oct3/4, Nanog, E-Ras, Cripto, Dax1, Zfp296, and Fgf4 for quality evaluation and smooth muscle actin, glial fibrillary acidic protein, and βIII tubulin for their differentiation potential [5]. When monitoring biomarkers, invasive sampling and measurement is widely accepted in preclinical research; however, this approach is often not suitable during the subsequent clinical stage of drug development. Extracellular substances, such as cytokines, hormones, and cellular metabolites, would be the most translatable biomarkers in cell culture supernatant, plasma and body fluids (urine, cerebrospinal fluid, saliva and tear). A typical example of the use of extracellular substances in pioneer research was c-peptide (insulin alternate) released in culture media upon glucose stimulation, as proof of functionality of iPS cell-derived pancreatic islet-like clusters [32]. Albumin secretion and urea production of iPS cell-derived hepatic cells were the other example of biomarkers in extracellular space [33]. Species differences must also be considered when translating preclinical biomarkers into clinical settings, particularly those regarding a drug’s effects on physiological and behavioral levels of specified targets. However, electrophysiological activities, such as measurement of cellular action potential of iPS-derived cardiomyocytes [34] and electrocardiography, would also work as a translatable biomarker. Standard clinical laboratory tests rely on easily measurable human biomarkers, but many of the biomarkers used during drug discovery and development processes are not well established. The acquisition and accumulation of fit-for-purpose clinical samples and data for biomarker validation require a long period of time [31]. Additionally, some clinical biomarkers, such as circulating tumor/rare cells, pathological changes (especially immune-related changes), nuclear medicine scans, behavioral/reflectional indexes, or psychiatric indexes, would not be applicable to iPS cell models. Therefore, it is essential to establish science-based strategies to overcome these issues at earlier stages of drug discovery. Ideally, a biomarker in clinical research will be an objective and noninvasive indicator of patient’s medical state that can be observed in an accurate and reproducible manner [30]. Selection of appropriate biomarkers and decision criteria is essential to provide evidence of the proof of concept (PoC) at earlier clinical development stages, such as Phase 1B (multiple ascending dose clinical trial in healthy volunteers) and 2A (pilot clinical trials to evaluate efficacy and safety in selected patients) [35]. A scoring system to assess the translatability of early drug discovery projects has been proposed [36]. Another scoring system to assess the translatability of early drug projects has been proposed [37] and applied to eight drugs that either were already approved for the market or had failed during the drug development process. Those scores highlight the importance of an accurate and reliable biomarker to reduce the risk of failure during drug development process [37].

## 3. Use of Induced Pluripotent Stem (iPS) Cells to Understand Diseases

The restricted availability and scarcity of patient samples has limited research progress, particularly in the field of rare diseases, in which both patient identification and sample acquisition are often extremely difficult. Therefore, immortalized cell lines and animal models have been used as alternatives. Importantly, cell lines established from a limited number of patients can often only give a glimpse into disease etiology. Moreover, the accuracy of disease modeling on the basis of animal models is compromised by interspecies differences [38]. In this respect, patient-derived human iPS cells allow researchers to generate an individualized patient iPS cell-derived “disease in a dish” [17], and isogenic (genetically identical other than mutation of a single gene) iPS cell lines provide an additional opportunity to study the intricate effects of gene mutations. After patient-derived iPS cells or their source (e.g., primary cultured fibroblasts and peripheral blood mononuclear cells) is obtained and banked, these cells can become an inexhaustible source of disease models. The first reported feasibility studies using patient-derived iPS cell-derived cells focused on Huntington’s and Parkinson’s diseases, diabetes mellitus and Down’s syndrome [11]. Shortly thereafter, the strategy to recapitulate disease phenotypes by using patient-derived iPS cells was applied to mimic spinal muscular atrophy (SMA) [39] and familial dysautonomia [40]. Currently, there are a substantial number of public, private and commercial human iPS cell banks containing pluripotent stem cell lines derived from normal donors and patients with various diseases. Owing to the immense interest in iPS cells, the repositories of these stem cell banks are still expanding. As we have already learned from human ES cell banking, the availability of well-characterized and quality-controlled iPS cell lines is essential to ensure that work from different laboratories can be replicated [41].

### 3.1. Disease Mechanisms

During a scientific inquiry, exploration of the disease mechanism generally is based on testable hypotheses. Through comparative testing based on -omics approaches comparing “normal vs. diseased” and “control vs. treated” profiles, the biological pathways that might be affected by a specific disease can be investigated. At present, iPS cells derived from patients with monogenic disorders genetically arising from single mutations are ideal systems for studying disease mechanisms and are an attractive platform for drug testing and drug discovery [42]. A systems biology approach combined with patient-derived iPS cells has vast potential for the investigation of diseases of unknown etiology, such as non-alcoholic steatohepatitis (NASH) [43]. However, a major concern surrounding both of these approaches is the feasibility of recapitulating the pathophysiological features of a disease state in vitro. From a practical standpoint, the exploration of various phenotypic differences between normal and disease states, such as morphology, cellular function or lipid accumulation, can provide scientists with clues that cumulatively lead to a better understanding of the disease. For example, neurons differentiated from Rett’s syndrome patient-derived iPS cells have been reported to show reductions in synapse and spine densities as a result of electrophysiological defects [44]. This iPS cell-derived neuron-based system has revealed that neuron-specific K(+)-Cl(−) co-transporter 2 (KCC2) is a critical component of the disease pathway [45]. Furthermore, the generation and clonal analysis of patient-derived iPS cells might be advantageous to elucidate unexpected somatic mosaicism, in which it is generally difficult to separate and analyze cells on the basis of the presence or absence of mutations. This advantage is exemplified by the use of iPS cells to recapitulate chronic infantile neurologic cutaneous and articular (CINCA) syndrome [46]. However, none of the disease models can entirely recapitulate the intricate interplay between a patient’s individual genetic background and the environmental factors that influence the disease development or the patient’s response to medication. Aging is another important factor to consider when investigating disease occurrence and progression in the laboratory settings. Recently, Ho et al. have applied transcriptome analysis to investigate the networks and pathways associated with spinal motor neuron (spMN) maturation and aging and have suggested strategies to further mature and age iPS cell-derived spMNs for amyotrophic lateral sclerosis (ALS) [47]. Because recapitulation of a disease phenotype is still a major technical hurdle, technological innovation in in vitro settings, such as three-dimensional cultures and “organ-on-a-chip,” are highly anticipated to be routinely incorporated in iPS cell-based disease modeling. The paradigm of disease understanding through use of patient-derived iPS cells is expanding, and the elucidation of the biological pathways that might be affected by specific diseases will provide opportunities to identify novel biomarkers not only for diagnosis but also for drug discovery.

### 3.2. Genetic Factors

Genome editing technologies were first applied to iPS cells for efficient generation of cell type-specific reporter lines, as well as tool lines with disruption, repair or overexpression of genes of interest [48]. Subsequently, the genome editing technologies have enabled the efficient generation of in vitro human iPS cell-based disease models by stably introducing genetic mutations of interest. At the earlier stage of method development for this approach, the significance of genotype-matched (isogenic) cell lines for disease modeling and drug discovery had already been pointed out in the study on laminopathy-associated LMNA mutations with patient-derived iPS cells [49]. Among the genome-editing technologies, CRISPR/Cas9 is one of the most promising systems; it can be efficiently applied in various research fields, including the elucidation of gene function, disease modeling and gene therapy. The CRISPR/Cas9 system has quickly become an area of special interest in generating isogenic human iPS cell lines [50]. Genome-wide association studies (GWAS) have indicated that various single-nucleotide polymorphisms (SNPs) can cause significant differences in susceptibility to disease development; however, to date, it has been difficult to obtain direct evidence supporting these observations. A major hurdle has been the sheer complexity of the genome and the estimation of the rate at which each SNP contributes to disease development and progression. Allele-biased gene expression identified in iPS cell-derived neurons might make genetic analysis more complicated [51]. In standard genetic approaches, samples from hundreds of patients are required for statistical analyses exploring the contribution of individual SNPs to the disease state. Isogenic human iPS cell pairs, which have the same genetic background except for the SNP of interest, may simplify these studies. Confirming the contribution of specific genetic variations to the development of a disease state provides scientists with clues as to how to develop companion diagnostics (see Section 3.4).

### 3.3. Hypothesis Confirmation in Clinical Settings

To confirm hypotheses of specific disease mechanisms in clinical settings, numerous ethical and technological limitations must first be overcome, as exemplified by difficulties in data analysis associated with samples generated from tissue biopsies and other interventional procedures. The identification and appropriate use of suitable and clinically feasible biomarkers is essential for the appropriate evaluation of a compound’s properties. This concept is translatable to both in vitro and in vivo iPS cell-based studies, in which a key goal is to identify appropriate biomarkers on the basis of a hypothetical disease pathway. Biomarkers in body fluids, such as plasma, urine, and cerebrospinal fluid, have been used by researchers because of the ease with which they can be accessed. In general, mRNAs, proteins, hormones, cytokines, and metabolites are measured by mapping these biomarkers to the landscape of the hypothetical disease pathway. For example, high susceptibility to oxidative stress was confirmed by quantitative proteomic analysis in iPS cells derived from a Huntington’s disease patient [52]. SOD1 (superoxide dismutase 1) and Prx (peroxiredoxin) families which were particularly affected in patient-derived iPS cells are expected to be clinical biomarkers in assessing efficiency of drug therapy. Circulating microvesicles/exosomes and specific microRNAs are also potent biomarkers with promising therapeutic applications in diseases such as multiple sclerosis [53] or liver injury [54]. Imaging, as well as physiological and functional measurements are also valuable biomarkers for hypothesis confirmation; however, assessing indicators usually requires a special set of skills and instruments. To prevent the delay or discontinuation of a study, the nomination and validation of biomarker candidates and the establishment of biomarker assays for clinical testing should be conducted at an earlier stage to verify the drug discovery and development processes (Figure 1B). If there is an approved medication that is known to act on a specific component of the pathway, an interventional approach might be feasible to confirm the hypothesis. Once clinically feasible and validated biomarkers related to a specific disease mechanism are identified, these biomarkers can also become valuable tools for diagnosis, patient recruitment and pharmacokinetics (PK)/pharmacodynamics (PD) studies (see Section 4.5). Aging of cells/tissues in the body is the other hurdle in understanding senescence-related or late-onset diseases, on their process and prevention. To shorten the development period for drugs targeting diseases such as Alzheimer’s disease and age-related macular degeneration, efficient use of iPS cell-based aging models is highly desired. One promising example is the application of progerin-induced aging of iPS cell-derived neurons, which resulted in induction of multiple aging-related markers and characteristics; including dopamine-specific phenotypes, such as neuromelanin accumulation. Furthermore, pathological changes including enlarged mitochondria or Lewy-body-precursor inclusions were observed in an iPS cell model for Parkinson’s disease [55].

### 3.4. Patient Stratification and Precision Medicine

**Figure 2:**
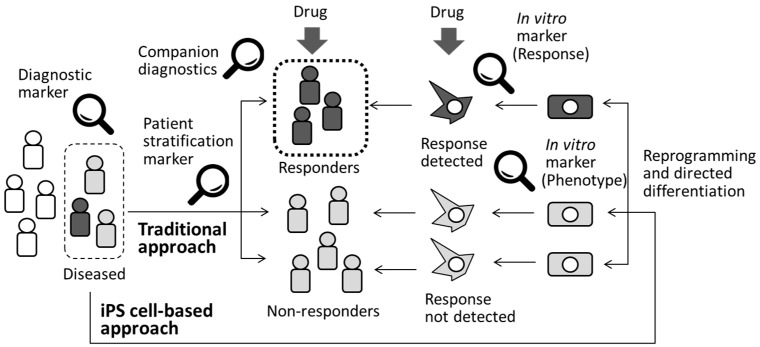
Diagnosis and patient stratification by using biomarkers. Application of iPS cell-based approach would be alternate way to identify drug responders and non-responders.

Although most of the established biomarkers routinely used for a definitive diagnosis at the clinical stage of drug development are called diagnostic biomarkers (Figure 2), heterogeneity among patients is a well-recognized factor that must be taken into consideration when designing clinical trials and during subsequent data interpretation. Pharmacogenomics is the research discipline that investigates the role of genetics in the drug response. Pharmacogenomic patient stratification involves examining drug responses in groups of patients selected for their specific genetic backgrounds and using genetic or phenotypic biomarkers [56]. These forms of tests are called companion diagnostics (Figure 2) and are developed at the clinical trial stage or even after a specific drug is released onto the market to enhance understanding of the potential toxicity or selectivity of a compound in a specific group of patients. Companion diagnostics is important not only to accelerate the process of drug development by providing an efficient clinical study design but also to facilitate precision medicine, which is the generation of a tailored medication for a specific group of patients. For example, iPS cells derived from malignant calmodulinopathic long QT syndrome patients with mutations involving CALM1, CALM2, or CALM3 mutations were used as tools to explore efficient therapeutic strategy for the disease [57]. Differential responses to lithium were detected in neurons from patients with bipolar disorder and they matched well with clinical outcomes [58]. Clonal analysis and directed differentiation of iPS cells from neonatal-onset multisystem inflammatory disease (NOMID) patients elucidated heterogeneity of monocytes from a single patient [59]. Subsequent identification of unexpected somatic mosaicism of NLRC4 mutation demonstrated the significance of prospective genetic screening, combined with iPS cell-based phenotypic dissection for individualized diagnoses. Therefore, an iPS cell-based approach would be a complementary approach to clinical testing to identify drug responders and non-responders, as shown in Figure 2. Recent advances in computational tools for analyzing large datasets continue to generate attractive hypotheses; however, patient recruitment for the verification studies requires years of effort. Therefore, the availability of multiple samples at a desired time point is another advantage of human iPS cell-derived disease models compared with standard preclinical models [60]. For this purpose, significant numbers of deposited iPS cell lines are required.

## 4. Pharmacological Studies Using iPS Cells

Currently, there are two distinct approaches for designing the drug discovery process: the “target-based” approach, which is focused on measuring changes to specified targets, and the more general “phenotypic” approach, which measures morphological changes in cellular phenotypes. Naturally, highly relevant human-derived cellular models recapitulating specific disease conditions are required for the proper identification of promising compounds, and iPS cells have emerged as one of the most promising tools for the reliable recapitulation of the disease state in vitro. The significant contribution of phenotypic screenings to the discovery of first-in-class small-molecule drugs has been reported [61]; however, the optimization process relies heavily on the identification of specific targets. As described below, the pharmacological part of drug discovery and development is a multi-step process, and each of the stages has specific points that must be considered.

### 4.1. Validation of Cellular Models

In vitro conditions differ significantly from the cellular environments found within the body because cultured cells are separated from most of the physiological regulatory systems. As noted in research on neurodegenerative disease models [62], cellular platforms suffer from a number of shortcomings at the in vitro stage of disease modeling. Therefore, any type of in vitro-based disease model must be carefully validated by specialists from various research areas. On the other hand, it would be more complicated to model and validate a cellular model for lifestyle-related diseases. Recapitulating key pathological features of metabolic disorders of the liver with iPS cell-derived hepatocytes of various inherited cases [63] might be an approach to better characterize common pathological changes of the liver in lifestyle-related diseases. In any type of pharmacological study, reference interventions, called “controls” or “standards,” are required. If there are established interventions or biomarkers to assess disease relevance, their use is prioritized. By being able to differentiate into specified cell types, patient-derived iPS cells, with either known or unknown genetic backgrounds, are undoubtedly one of the most advantageous tools to recapitulate disease-associated molecular events in vitro. Moreover, recent advances in gene manipulation technologies have played a significant role in the establishment of human iPS cell-derived cellular models with specific and disease-relevant genetic variations (see Section 3.2). Importantly, genetic manipulation of a singular gene in iPS cell-based disease modeling should be interpreted cautiously because most diseases are caused by an intricate combination of genetic, physiological, environmental, and lifestyle factors, many of which have not yet been well elucidated. The addition of substances such as cellular stress inducers that aim to alter the physiological status might be useful to recapitulate or enhance a cellular disease phenotype; however, the subsequent data interpretation should be carefully assessed for the relevancy of the selected cellular stress inducers to the pathophysiological and spatiotemporal events in patients. Moreover, in most cases, it is difficult to monitor phenotypic changes in patients’ target cells because these cells are difficult to access. Therefore, surrogate biomarkers that are selected to substitute for this shortcoming are an essential part of TR. These surrogate biomarkers can be exemplified by cellular products/metabolites, exosomes, and appropriate probes for positron emission tomography (PET) imaging, and can be applied in TR; however, their discovery and development often occur after the identification of drug candidates. Without highly relevant biomarkers that can be used to assess the pharmacological effects of drug candidates in patients, the evaluation of a drug’s proof-of mechanism (POM) is hampered. Therefore, the careful validation of cellular models is crucial not only to recapitulate the disease state in a dish but also to subsequently translate the anticipated results. Thus, both the disease-relevancy and translatability, particularly the availability of biomarkers and reference interventions, should be assessed before the initiation of a drug discovery project using human iPS cell-derived models.

### 4.2. Screening and Pharmacological Evaluation of Drug Candidates

During the “hit-to-lead” phase of drug development, numerous hit compounds are identified from a pre-defined compound library by using a high-throughput screening (HTS) approach and subsequent selection and optimization of several promising lead molecules. The application of HTS techniques, assay sensitivity and reproducibility are prerequisites for hit compound identification at this stage [64]. High-content screening (HCS) is another powerful tool that is widely used in drug discovery. This multiplex assay is focused on analyzing whole cells or their sub-cellular components and providing a simultaneous readout of numerous parameters [65]. In general, after the selected cell types are incubated with test compounds, HTS is applied for target-based screening, whereas HCS aims at detecting phenotypic changes. Measurable traits applied at this stage are sometimes referred to as biomarkers; however, many of these indicators are not applicable at the subsequent clinical stage of drug development as discussed in Section 2. A recent review article by Tang et al. has summarized the current status of disease models and compound screening with patient-derived iPS cells [66]. A list of hit compounds (referred to as rescue agents) that are used in iPS cell-based compound screening assays include clinical chemicals (FDA-approved or passed a Phase 1 clinical trial), a few of which, after showing positive effects on disease phenotypes, are expected to bypass the early stages of drug development. However, dose-response relationships and selectivity are other points to consider when evaluating which drug candidates have the potential to become lead molecules. After careful assessment of the baseline, the maximum response, the time course of the response, the half maximal effective concentration (EC50) and the half maximal inhibitory concentration (IC50) are calculated as a measure of a drug’s potency [67]. The difference between the effective dose and the dose that causes side effects is called the safety margin, and a wide margin is desirable when considering drug repositioning. Therefore, at this stage, numerous hit molecules might be used as probes for further exploration of the lead compound’s target or its mechanism-of-action (MOA) rather than as lead molecules themselves.

### 4.3. Target Identification and Validation

If a drug candidate is identified through phenotypic screening, the subsequent verification of its target [68] and MOA [69] is required for the early prediction of its pharmacological and toxic effects on animal model systems. In general, drugs fail at the clinical stage for two main reasons: the lack of efficacy and/or safety issues [70]. Efficacy is the capacity of a compound to have a significant therapeutic effect in an experimental setting; however, there are multiple factors, including interspecies differences that often prevent the proper evaluation of a compound. Therefore, the assessment of a compound’s efficacy in Phase 2 clinical trials is still an indispensable and final step required to confirm its therapeutic effects in patients. However, target identification would also allow a shift from phenotype to target-based drug discovery, and HTS can be used to identify drug candidates with higher efficacy [68]. For example, a large-scale phenotypic screen with neurons from Fragile X syndrome (FXS) patient-derived iPS cells resulted in the identification of several compounds with weak activity to reverse disease phenotype [71]. The potency of the compounds were not sufficient for medical application but they were expected to be useful probes for setting up large-scale screening and in vivo studies to find drug candidates or identification of target molecules underlying the mechanism of FXS. Safety issues are preferred to be addressed before clinical studies are conducted, to ensure the safety of the participants. Adverse or toxic effects are elicited by either target molecules or downstream pathways (on-target) or other known or unknown mechanisms unrelated to the target molecule (off-target). In general, the compounds and cytokines used to induce the differentiation into cell types of interest are well validated for their interaction with the corresponding targets. However, kinase inhibitors, for example, show variable degrees of selectivity or promiscuity within their family [72]. Therefore, it is essential to confirm a compound’s effect on a target molecule as specifically as possible at both the preclinical stage, by utilizing numerous pharmacological models, and the preclinical evaluation phase, in patients.

### 4.4. Target Engagement

**Figure 3:**
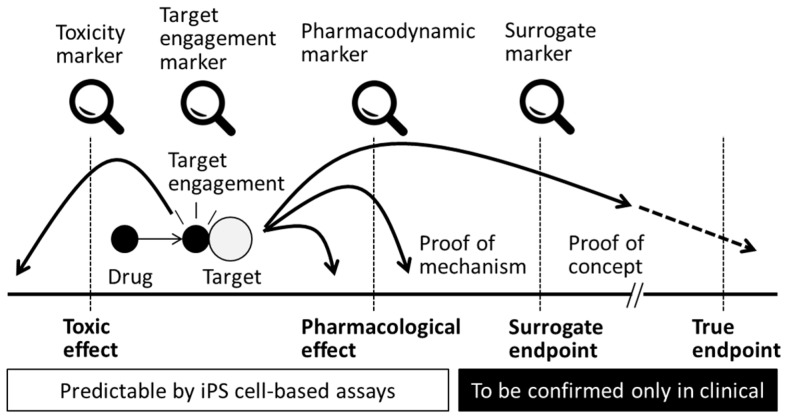
Biomarkers in efficacy and safety assessments. Application of in vitro assessment with iPS cell-based assay would be able to predict success or risk of a drug discovery project at an earlier stage.

At the target engagement stage of drug development, lead drug candidates are evaluated on the basis of the specificity with which each compound interacts with its intended target molecule at the appropriate concentration [73] and whether it affects the proximal site of the expected biological pathway in a living system. The metrics used at this stage are generally called the “target engagement biomarkers” (Figure 3), and a wide variety of methods are available to measure these indicators. Positron emission tomography (PET) imaging is one of the most popular technologies for target engagement in the field of translational medicine because this method is non-invasive. For example, PET imaging was applied to confirm survival of iPS cell-derived dopaminergic neurons after transplantation [74], but some of the probes used were originally developed for target engagement of dopamine-related molecules. PET probes can provide numerical data on a target’s activity or any target-drug interaction. In that sense, in vitro-based studies can also be applied to evaluate target engagement. For example, cholinergic and dopaminergic neurons are target cells in Alzheimer’s and Parkinson’s disease drug discovery, respectively, and medications are expected to induce drug-target interactions or physiological changes in target cells. If target engagement is not confirmed for a drug candidate, and the drug fails to produce an expected pharmacological result, it is then very difficult to evaluate the reasons for the drug’s lack of efficacy. Because the establishment of a target engagement assay itself is difficult and time consuming, it is preferable to incorporate target-based research as early as possible during the drug development process.

### 4.5. Pharmacokinetics and Pharmacodynamics

Pharmacokinetics (PK) is a research discipline that is focused on evaluating the time course of absorption, distribution, metabolism, and excretion (ADME) of an administered drug. Pharmacodynamics (PD) investigates the relationship between a drug concentration at the site of action and the resulting effect, which is monitored by using a set of biomarkers (PD marker, Figure 3) and considering the time course and intensity of the therapeutic effects. In general, the parameters of ADME are not considered in in vitro studies except for the permeability of cellular membrane assays because, in the in vitro environment, cells are exposed to compounds or growth factors that are directly added to the culture medium. In the in vitro setting, the dose-response relationship and time course of pharmacological effects can be monitored; however, these data cannot be directly extrapolated to in vivo studies. Preclinical PK/PD studies are an indispensable step in the design of clinical studies because they investigate the dose-response relationship prediction and dosing regimen in living organisms to maximize the lead compound’s therapeutic benefits while reducing its toxic effects [75]. To meet the stringent regulatory requirements, the PK/PD stage of drug development requires both significant time and monetary investment; therefore, the early consideration of in vivo models, biomarkers and their relevancy to the clinical setting is highly preferable.

In vitro ADME screening with fit-for-purpose models is another important discipline of drug discovery. Primary human hepatocytes are effective tools for the in vitro evaluation of metabolism, drug-drug interactions, hepatotoxicity, and transporter activity. The recent advances, the potential, and the remaining issues underlying the generation of hepatocytes from human iPS cells are discussed in the toxicology part of this review (see Section 5.1). Apart from primary hepatocytes, other primary cell types of interest in ADME analyses are cells of the intestinal wall (adsorption), blood-brain barrier (BBB, transportation) and kidney (excretion); however, the establishment of corresponding in vitro models has been extremely difficult. Recent advances in the differentiation of human iPS cells into a variety of different cell types have enabled preparation of functional enterocyte-like cells [76], BBB endothelial cells [77,78] and renal tubules [79]. The standardization of these iPS cell-derived systems to meet the regulatory requirements of various NCE-approving agencies will be the next challenge for this promising research tool.

### 4.6. Clinical Endpoints

The purpose of medication is to cure a disease or to remedy a condition. For example, medications are often intended to decrease symptoms, improve impaired function, extend the disease-free interval and prolong life. The criteria with which medications are characterized are defined as the true endpoints or primary clinical endpoints (Figure 3). At the clinical stage of drug development, the surrogate endpoint is a biomarker that measures the effectiveness of developed drugs when the primary endpoints are undesired (e.g., worsening of the condition or death) or is represented by an insignificant number of measurable traits (Figure 3). Surrogate markers that correlate well with the primary clinical endpoints are preferred; however, to date, the relationship between these two endpoint measures has not been firmly established. Confirmation of true or surrogate endpoints needs to be achieved in the clinical stage, but nomination of surrogate markers would be feasible through preclinical research with iPS cells, especially with disease mechanisms. For example, pharmacological evaluation of tyrosine kinase inhibitors with CD34+ cells derived from 8p11 myeloproliferative syndrome patient-derived iPS cells identified a reduction in the number of colony forming units (CFUs) of CD34+ cells and a similar effect was observed in peripheral blood cells of patients [80]. In this case, CFU of CD34+ cell was a candidate for a surrogate endpoint in a clinical evaluation. Surrogate markers can be exemplified by glycosylated hemoglobin, which is used to predict diabetic complications or plasma cholesterol levels for the determination of cardiovascular events; however, the predictability of most of these biomarkers is still under discussion, although they are being used in many clinical trials. Determining a more stringent set of criteria for surrogate endpoint markers is beneficial to reduce both the duration and the size of clinical trials. These biomarkers would then be more reliable in the drug approval process. It is also important to bear in mind that the selection and use of inappropriate surrogate endpoints can lead to the generation of incorrect conclusions, which in turn may lead to the release of medications that have inefficient or undesired effects. In that sense, surrogate endpoints would be most predictive when the pathophysiology of the disease and the mechanism of action of the medical intervention are thoroughly understood [81]. Because all of the points discussed above are directly applicable to drug candidates that can be identified with iPS cells, it is desired to establish and apply clinical endpoints and corresponding biomarkers at earlier stages of drug development.

### 4.7. Integrative Approach

When the toxicological effects of lead compounds on organs, such as the kidney, intestine and skin, are detected in general toxicology studies based on animal models, iPS cell-derived models are valuable platforms for the confirmation and mechanistic analysis of these compounds in humans at the preclinical stage of drug development. A major issue of the utilization of iPS cell-derived cells is the necessity of first generating numerous and functional cell types organized as tissues rather than typical in vitro two-dimensional cultures. Recently, “tissue chip” devices have become prominent because these cellular models are designed to recapitulate the structure and function of human organs, such as the lung, liver and heart; therefore, they are often called “organs-on-a-chip” or “human-on-a-chip” [82]. Three-dimensional model systems are expected to be able to mimic human physiology more accurately than traditional two-dimensional cultures, and “organoid-on-a-chip” and “body-on-a-chip” are advanced formats of “tissue-on-a-chip” devices [83]. The goal of these in vitro advances is to enable researchers to investigate the potential effects of a test substance in the same manner as would be used in a Phase 1 study. Once “tissue-on-a-chip” devices are developed and accepted as a standard part of the preclinical drug development process, they can be used to predict whether a drug candidate is safe or toxic to humans in the safety of the laboratory setting.

### 4.8. Benefits of iPS Cells in the Toxicological Assessment of Drugs Targeting Rare Diseases

Because the number of patients with rare diseases is limited, it is often impossible to conduct Phase 2 trials that would adhere to the regulatory scale and format typical for common diseases. Patients suffering from rare diseases hope to access new medications as quickly as possible; however, evaluating the safety of newly developed compounds is especially difficult in such cases. Therefore, through the concomitant use of patient-derived iPS cells to access the efficacy and safety of a compound via the “disease in the dish” approach, it may soon be possible to advance the prediction of a compound’s pharmacological and toxicological effects in the clinical setting.

## 5. Cytotoxicity

Given that unforeseen toxicity is frequently detected at the costly clinical stage, there is a clear need to bridge the gap between preclinical in vitro toxicology testing and establishment of biomarker assays to be utilized in the clinical phase of drug development. One way to achieve this type of assessment is by applying a similar set of established and robust biomarkers and evaluating the response similarity between both environments. This approach continuously proves to be difficult because most of the in vitro preclinical and clinical toxicology assays rely on variable and non-overlapping cellular models. In vitro preclinical toxicity testing relies on the use of immortalized and often irrelevant cell lines. In vivo animal testing is the primary method and is crucial for the preclinical prediction of toxicity because it offers necessary insight into the biological aspects of ADME. However, the differences between animal models and humans and the limited scalability make it difficult to develop and rely on preclinical biomarkers to accurately predict toxic effects at the clinical stage of drug development [84]. Owing to their capacity to recapitulate the donor’s phenotype, iPS cells have the potential to bridge preclinical and clinical in vitro toxicity testing by generating human cellular derivatives in which the same or highly relevant sets of biomarkers can be applied throughout the drug discovery and development process (Table 1). Despite the fact that iPSC-derivatives still represent fetal rather than the adult stage of development and maturation, with the rapid progression of iPS cell differentiation protocols, it is only a matter of time before iPS cell derivatives match the maturity and functionality of primary human cells and pave the way for the use of assay-dependent biomarkers for in vitro predicative toxicology at any given stage of the drug development process.

### 5.1. Hepatotoxicity

The architecture of the liver has evolved to metabolize a wide range of organic substances, including drugs, which enter an organism from the environment. As some of these substances can be harmful, the role of hepatocyte-orchestrated xenobiotic metabolism is to make them more soluble and therefore easily excreted. Species-dependent mechanisms of xenobiotic detoxification, as well as a high degree of inter-individual variability affecting human drug metabolism, make it difficult to predict hepatotoxicity of a potentially promising compound before administrating it at the clinical stage of drug development. As a consequence, unforeseen hepatotoxicity is one of the main reasons for drug attrition at all phases of clinical development and for the withdrawal of drugs already released onto the market [85]. At the preclinical stage of drug development, the results from animal models often inadequately reflect the intrinsic and idiosyncratic hepatotoxicity that can later emerge in human trials [86,87]. On the other hand, in vitro models for predictive cytotoxicity at the very initial stage of drug development rely heavily on first immortalized sub-optimal cell lines like HepG2 or Huh-7 cells. Assays performed at this point are focused on measuring cellular viability, but as these easily expandable cells suffer from a very limited or non-existent hepatic functionality that is needed for proper xenobiotic metabolism [88], some of the toxic compounds remain in the drug discovery process. An interesting exception among immortalized hepatic cell lines are HepaRG cells which retain a high level of metabolically active hepatic enzymes. Nonetheless, this cell line represents only a singular donor, its functionality depends on a prolonged incubation with 2% dimethyl sulfoxide [89,90] and cells are not efficiently expanded. As the in vitro stage of drug development progresses toward the chemotype selection of potential drug candidates and subsequent optimization utilizing drug metabolism and pharmacokinetic (DMPK) parameters, primary hepatocytes have become an obvious choice for the detailed in vitro prediction of hepatotoxicity. Primary hepatocyte-based in vitro multi-assay panels investigating mechanisms by which compounds can cause drug-induced liver injury (DILI) are often focused on measuring changes in hepatic CYP induction, reactive metabolite formation, mitochondrial dysfunction and inhibition of the bile salt export pump (BSEP). However, owing to high donor-to-donor variation with an easily exhaustible source of specific donor cells and difficulties associated with maintaining cellular functionality in vitro, more specialized and continuously available platforms to evaluate hepatotoxicity are still sought [91]. At present, iPS cell-derived hepatocytes still represent an immature rather than an adult stage of hepatocyte development, as evidenced by the expression of high levels of fetal markers, such as AFP and DLK-1 [92,93]. More importantly, one of the most desirable characteristics needed for improving translational prospects of these cells is sustained functionality of a handful of the most important hepatic cytochrome P450 enzymes, since these CYP proteins are involved in metabolism and bioactivation of more than 75% of drugs [94]. Despite the fact that human PSC-derived hepatocytes are still characterized by lower CYP enzymatic activity when compared to fresh primary hepatocytes, these cells have already been demonstrated to be a valuable platform for hepatocyte-specific toxicity assays [95,96,97,98] (Table 1). Moreover, iPSC-derived hepatocytes exhibit high levels of other hepatic functions, such as synthesis and secretion of various plasma proteins (carrier proteins like albumin, blood clotting factors and apolipoproteins), glycogen storage, urea production, and bile production. With rapidly improving hepatic characteristics, stem cell-derived hepatocytes may also soon find a place in the preclinical stage of drug development as a source of cells to detect changes in the secretion of common clinical markers of DILI, e.g., alanine aminotransferase (ALT) [99]. Compared to any other hepatic cell types used at various stages of drug discovery, iPS cell-derived hepatocytes have the advantage of being able to mimic the genetic backgrounds of patients who present with idiosyncratic DILI or subsequent adverse drug reactions (ADR) after the administration of a compound. Therefore, iPS cell-derived hepatocytes can become a tool for retrospective clinical data analysis. Because patient-derived iPS cell lines can be generated from blood samples, it is possible to derive iPS cell-derived hepatocytes specifically from patients who present with DILI during clinical trials or who took a drug that was already approved for market release.

From the perspective of bridging preclinical and clinical biomarker use, which is in contrast to most immortalized hepatic cell lines, stem cell-derived hepatic cells, primary hepatocytes, and HepaRG cells express high levels of the microRNA miR-122, which is rapidly becoming a highly relevant and easily translatable marker of hepatotoxicity. MiR-122 constitutes approximately 70% of all microRNAs expressed in hepatocytes, and an increase in circulating miR-122 in serum plasma has been shown to correlate with and even precede the typical increase in ALT levels after DILI [100]. Interestingly, increased level of circulating hepatic miR-122 was reported to correlate well with the severity of progression of non-alcoholic fatty liver disease (NAFLD) and non-alcoholic steatohepatitis (NASH) [54,101,102].

Regardless of hepatic cell type used at the in vitro stage of drug development, cell culture environment has a major impact on cellular behavior and functionality, and therefore improvements in how scientists model the in vivo environment when using iPS cell-derived hepatocytes in vitro are important to bridge preclinical and clinical biomarker utilization. Continuous expansion of the number of groups working on human PSC-based hepatocyte protocols has produced a variety of methods for generation of hepatic cells, with recent ones leading towards three-dimensional single or co-culture systems [103,104,105,106]. Interestingly, in 2016, McCarthy et al. reported on the generation of a primary hepatocyte-based liver-on-a-chip microfluidic device in which spatially controlled zonation found within the liver lobules and the subsequent zone-dependent metabolism are maintained [107]. Improved stem cell differentiation methods, combined with the likes of a complex culture systems, should soon lead to the generation of patient-derived, metabolically functional “human-on-a-chip” models for the prediction of hepatotoxicity before a drug is given to a patient [108,109].

### 5.2. Cardiotoxicity

Unforeseen cardiotoxicity is one of the leading causes of drug attrition at the most expensive stage of drug development (Stage 3 of clinical trials) and can also be responsible for adverse drug reactions related to arrhythmias and post-release drug withdrawal from the market [21]. This issue is particularly true for cancer therapeutics that aim to inhibit kinases that are overexpressed in the cancerous state. Modulation of these kinase pathways is often associated with off-target cardiotoxicity because the same kinases are also expressed by the heart cells [91]. Although animal studies play an important part in predicting the cardiotoxicity of drug candidates, differences in certain electrophysiological properties between animals and humans can often limit the translation of the results to the clinical setting [110,111]. Moreover, the detection of hERG inhibition in vitro relies heavily on mouse and human cardiac muscle cell lines [112] and non-cardiac cell lines, such as CHO or HEK293. The main desirable characteristic of these cells is the overexpression of the hERG gene encoding inward rectifying voltage-gated potassium channels, which are involved in cardiac repolarization [113]. All lead compounds must be assessed for their potentially toxic effects on the prolongation of the QT intervals through the blockade of the hERG channel and the release of cardiac troponin I and T, and iPS cell-derived cardiomyocytes have already been demonstrated to be a valuable source for these assays [114]. Although iPS cell-derived cardiomyocytes still represent the fetal stage of cardiac development, these cells already express many of the ion channels encoding genes necessary for the proper function of an adult human heart, thus making these cells a desirable tool to screen for cytotoxic effects on cardiac ion channels [91]. In 2013, Liang et al. reported the generation of iPS cell-derived cardiomyocytes from healthy subjects and from individuals with hereditary QT syndrome, hypertrophic cardiomyopathy and dilated cardiomyopathy (Table 1). The authors have shown that this iPS cell-cardiac cell panel predicts susceptibility to cardiotoxic compounds more accurately than a standard hERG gene-based in vitro assay using the HEK293 cell line [115]. Interestingly, the heterogeneous nature of emerging iPS cell-cardiomyocytes can be overcome by microRNA switch-based purification systems [116]. These defined populations of iPS cell-cardiomyocytes in a pre-designed three-dimensional system closely recapitulating human cardiac tissue have the potential to become an indispensable tool for the discovery of cardiotoxicity biomarkers in a relevant and translatable manner.

### 5.3. Nephrotoxicity

Under normal conditions, the kidneys filter roughly 150–180 L of plasma every 24 h [117] and receive around 25% of cardiac output, rendering them, as with the liver, naturally susceptible to drug-induced injury [118]. Podocytes in the renal corpuscle and renal proximal tubular cells are usually most affected [119]. Perturbation of normal kidney function can lead to an accumulation of waste products resulting in acute kidney injury (AKI), long-term chronic kidney disease (CKD) or progression to the end-stage renal injury (ESRI). Unfortunately, damage to the kidneys is often detected late because commonly used standard clinical biomarkers, such as serum creatinine (sCr) and blood urea nitrogen (BUN), have limited sensitivity and specificity for nephrotoxicity [120]. All of these factors make it difficult to predict nephrotoxicity during the drug development process. In fact, at the preclinical stage, it has been estimated that only approximately 7% of new drug candidates fail because of nephrotoxicity [121], thus highlighting certain translational discrepancies between preclinical and clinical observations. In vivo nonclinical nephrotoxicity assessment is often focused on administering lead compounds to rodents and large animals, such as dogs and monkeys [122], and subsequently measuring of standard biomarkers, including BUN and sCr, with postmortem histology. The evaluation of in vivo nephrotoxicity could be supplemented with additional biomarkers, including a promising proximal tubule injury biomarker (KIM-1), neutrophil gelatinase-associated lipocalin (NGAL), and cytokines such as macrophage colony-stimulating factor (M-CSF), interleukin-6 (IL-6), and IL-8 [123,124]. However, owing to interspecies variability, animal models still inadequately predict subsequent reactions in humans. On the other hand, in vitro systems do not reflect the complexity of kidney tissue and it is therefore difficult to bridge in vitro to in vivo modeling of nephrotoxicity. Moreover, typical MTT and resazurin cytotoxicity assays performed in in vitro models fail to detect nephrotoxicants in a clinically relevant manner [123,124]. However, due to the increasing rate of adverse drug reactions, numerous immortalized kidney cell lines and primary cell lines are often used to complement in vivo studies in assessing early nephrotoxicity effects of lead compounds. Among the most commonly used renal cell lines are human HK-2 and HEK-293, rat NRK-52, monkey JTC-12, dog MDCK, and mouse BUMPT-306 proximal tubular cell line. Among primary renal cells, these include commercially available human renal proximal tubule cells (HRPTEC) which are isolated from the proximal tubule, renal epithelial cells (HRE) from the epithelial cells of the cortex and glomerular, renal cortical cells (HRCE) comprised of both proximal and distal tubule cells), and normal human mesangial cells (NHMC) [125]. However, these valuable in vitro cell models suffer from the common issues associated with intra-individual variability, an easily exhaustible source of donor cells, detrimental effects of cell culture conditions on cellular functionality, and from possible inconsistencies during the isolation procedure. As in vitro preclinical systems do not reflect the complexity of the kidney organ, it is therefore difficult to bridge this stage of drug development to in vivo toxicity studies. In the face of these obstacles, human iPS cell-derived nephron cells are quickly becoming an interesting source of cells for the early detection of nephrotoxicity and disease models. In a 2015 study by Huang et al. the authors exposed a commercially available immortalized human HK-2 cell line and primary cell HRPTEC as well as in-house generated fresh primary hPT (human renal proximal tubule) epithelial cells to six known nephrotoxins and evaluated the increase of in kidney injury biomarkers KIM-1, NGAL and M-CSF [123]. The authors reported that these markers were not suitable for the in vitro evaluation of nephrotoxicity in the immortalized human HK-2 cell line; however the expression of both KIM-1 and NGAL was upregulated upon stimulation with known nephrotoxins in both primary renal cell types. This study further highlighted that cellular models based on primary cells are superior to common biomarkers of nephrotoxicity detection used during the in vivo stage of preclinical drug development. In 2015, Kandasamy et al. proposed a simplified eight-day protocol for differentiation of induced pluripotent stem cells to renal proximal tubule cells (HPTC) by culturing iPSC in renal epithelial cell growth medium (REGM) supplemented with two bone morphogenetic proteins, namely BMP2 and BMP7. Although certain stemness markers certainly require further downregulation, the response of emerging hiPSC-derived HPTC-like cells to acarbose, ethylene glycol, and potential nephrotoxins aristolochic acid, and cisplatin in the IL6/IL8 assay was highly coordinated with that of the primary HPTCs [119]. Most of the iPSC differentiation protocols, however, aim at three-dimensional derivation of mini kidney organs that would more closely recapitulate an intricate nephron structure [126,127]. Takasato et al. reported differentiation of human ES cells to simplified self-organizing three-dimensional kidney structures comprised of early nephrons with their potential use in the nephrotoxicity studies [128]. Further advancement to the protocol came a year later from the same group of scientists, when they reported generation of kidney organoids comprised of multiple cell types. The authors managed to induce cellular prevalence to differentiate among nephrons segmented to distal and proximal tubules, early loops of Henle as well as to glomeruli comprised of podocytes. Transcriptional profiling of these kidney organoids was estimated to correspond to the first-trimester human kidney. Moreover, when differentiated for 17 days, organoids were exposed for 24 h to known nephrotoxin cisplatin; proximal tubule cells within these organoids underwent apoptosis in a concentration-dependent manner [79]. Another example of applying iPSC-derived self-organizing kidney organoids for the nephrotoxicity evaluation was reported in 2015 by Morizane et al. [129]. After 21 days of differentiation, kidney organoids were exposed for 48 h to 5 mg/mL of antibiotic gentamicin or for 24 h to 5 μM of cisplatin. Organoids were then stained for the in vivo biomarker of kidney injury KIM-1. To mark proximal and distal tubules, cells were respectively co-stained for LTL and E-cadherin. Interestingly, KIM-1 was expressed in LTL positive cells but not in E-cadherin positive cells, therefore highlighting the relevance of this in vitro organoid system to the in vivo settings. With kidney-on-a-chip systems gaining considerable interest for the establishment of more physiologically relevant 3D systems [130,131] and with the current fast-paced optimization of human iPS differentiation protocols, it is just a matter of time before these two technologies will be combined to further aid in the optimization of preclinical nephrotoxicity evaluation.

### 5.4. Neurotoxicity

The preclinical evaluation of neurotoxicity is mainly comprised of costly in vivo animal experiments; however, the difference between human and animal models and the lack of screening scalability present major obstacles in translating these results to clinical settings. The majority of relevant in vitro assays are based on rat primary cortical models [132] and tumor-derived neuronal cell lines, which present limited usability, owing to the sheer complexity of the human central and peripheral nervous system. Moreover, in vitro neurotoxicity assays often focus on the detection of general cytotoxicity rather than targeting neuronal- and glial-specific toxicity. The role of BBB cells, highly specialized endothelial cells that limit the accessibility of the brain to circulating compounds, is also of crucial importance in the in vitro detection of neurotoxicity [133,134,135]. As compounds with a molecular mass over 500 Da cannot pass through the BBB, only a small percentage of molecules affect the central nervous system. The differences in the BBB between humans and in vivo animal models make it difficult to accurately translate preclinical biomarkers, such as P-glycoprotein (P-gp), claudins and transporters, to the human trial stage [135]. Because of these limitations, pluripotent stem cell-derived neurons, endothelial cells, astrocytes, glial cells, and smooth muscle cells have prominent roles in the detection of neurotoxicity in vitro [136,137] and the identification of translatable biomarkers. In a 2016 study [132], the spontaneous neuronal activity of commercially available iPS cell-derived neurons has been shown to be modulated by toxicological and pharmacological compounds. The co-culture of iPS cell-derived neurons with astrocytes improves sensitivity to neurotoxins. Further improvement of bursting in these still-immature cells should cement the role of iPS cell-derived neurons in translating in vitro neurotoxicity findings into new medical treatments.

## 6. Cell Therapy Using iPS Cells

In this review, the application of iPS cell derivatives in the field of cell therapy is not the main focus; however, it is worth mentioning that incorporating certain elements of TR at early stages of clinical study design would be beneficial to predict and avoid the potential pitfalls of a research program. For example, an impact evaluation of cell-based therapies should depend on the number of viable iPS cell-derived cells that survive transplantation rather than on the initial number of administered iPS cell derivatives. However, post-transplantation monitoring of surviving cells is problematic, particularly in the clinical setting, which is why the first allogenic iPS cell-based cell therapy trials focused on macular degeneration of the retina, where the fate of the transplanted cells is relatively easy to follow [138]. Nonetheless, the co-development of biomarkers to monitor/track cells, such as enzyme-linked immunosorbent assays (ELISAs) for cellular products and imaging tracers, would be essential for dose setting, efficacy assessment and long-term monitoring.

## 7. Future Perspectives

The most advantageous feature of human iPS cells in drug discovery is the capacity of these cells to recapitulate the donor’s phenotype in vitro, thus ushering in an era of “clinical trials in a dish” [139]. The growing number of human iPS cell banks that are being established worldwide confirms various research organizations’ unflagging interest in iPS cells. However, the important point that must be kept in mind is that these banks usually include a sample of subjects; therefore, the results of these studies should be carefully validated before they are generalized to an entire population [140]. The knowledge and understanding of modeling diseases using human iPS cells and applying iPS cells during the drug discovery and development process are still not well established except for in the toxicology field. Previously, we developed a simple protocol to generate insulin-producing cells from human iPS cells [141]. Our research focus was the use of these beta cell-like cells as a screening platform for the identification of new compounds with novel modalities and as a source of cells for cell therapy. Moreover, Baden et al. have reported modeling viral infections in human pancreatic β cells by using the same technological platform [142]. This model is a good example of different iPS cell-based approaches that ultimately have similar aims to improve therapeutic outcomes. Although continuous efforts have been made to apply human iPS cells in a variety of processes exploring disease understanding and improving drug discovery, the approach to iPS cell-based research needs to be more integrative and collaborative.

At the clinical stage of the therapeutic evaluation of new medicines, it is essential to select an appropriate set of biomarkers [35], and, as discussed in this review, these biomarkers should also be applicable in the preclinical phase of drug development. Notably, the Human Genome Project has generated an unprecedented amount of information regarding the structure and function of genes, with proteomics, metabolomics and other -omics disciplines following suit. As research data grow at an exponential rate, the application of systems biology [43] and computational and mathematical modeling of complex biological systems to iPS cell-based disease understanding and drug discovery is the next challenge. The integration of each building block with the entire process is the direction in which the drug discovery process that incorporates human iPS cells should be moving.

## 8. Conclusions

Derivation of human induced pluripotent stem (iPS) cells has revolutionized the fields of regenerative medicine and translational science, bringing a variety of exciting promises including an improved process of biomarker discovery, generation of advanced, highly relevant cellular platforms for early detection of compound-induced toxicity, as well as new treatments to patients suffering from rare diseases. One of the key challenges that remains to be overcome before iPSC-derived cells can be fully utilized in the drug and biomarker discovery process is the elucidation and fine-tuning of mechanisms involved in cellular maturation and sustained functionality. In fact, with each passing year, more sophisticated and robust human embryonic stem (ES) and iPSC-based protocols are being published, highlighting the necessity of providing the derivatives of these cells not only with a set of stage-specific developmental signals, but also with the environment more closely recapitulating the cellular state in vivo. Since human iPS cells were derived merely a decade ago, the dynamic expansion of iPSC-based research is simply astonishing, and further improvement of the differentiation methods will certainly bridge various aspects of basic and clinical research. We hope that this review will help those who are interested in exploring the potential of iPS cells to grasp the full picture of drug discovery and development.

## Figures and Tables

**Table 1:** Human iPSC-derived cells as an in vitro screening platform for detection of drug-induced toxicity.

	Author/Year/Title	Expression of Stage-Specific Markers in Human iPS Cells	Toxic Compounds Tested	Biomarkers Tested	Comments
Hepatotoxicity	Ware et al. 2015 [98]; Prediction of Drug-Induced Liver Injury in Micropatterned Co-cultures Containing iPSC-Derived Human Hepatocytes	CYP3A4 activity corresponding to ~90% of primary hepatocytes cultured for 24 h in vitro Albumin to alpha-fetoprotein ratio 12.2 at day 21 of culture	47 compounds were segregated into three groups based on previous study performed on primary hepatocytes (hepatotoxic, non-hepatotoxic, and compounds previously incorrectly classified as non-toxic).	Albumin secretion (ELISA); Urea production	Micropatterned co-culture system of iPSC-hepatocytes with fibroblasts prolonged liver hepatic functions up to 4 weeks when compared with single culture condition. Co-cultured micropatterned hepatic cells showed predictive DILI capabilities of 65-70% and 100% for sensitivity and specificity, respectively. Changes in urea production was the most sensitive assay endpoint
	Tasnim et al. 2016 [97]; Functionally Enhanced Human Stem Cell Derived Hepatocytes in Galactosylated Cellulosic Sponges for Hepatotoxicity Testing	AFP, ALB, AAT, HNF4a, CYP3A4, CYP3A7, CYP1A1, CYP1A2, ASGPR, MRP2 (qRT-PCR) Urea and Albumin production CYP induction (LC-MS)	APAP, Troglitazone, Methotrexate (24 h exposure)	Cell viability	Cellulosic scaffolds used during final stage of maturation enhanced hPSC-hepatocyte functions, including CYP activity and sensitivity to methotrexate Expression of alpha-fetoprotein was higher than albumin at Day 20 and 32 in both 2D and 3D cultures, however the albumin to alpha-fetoprotein ratio was the highest at days 32 in the 3D cultured
Cardiotoxicity	Liang et al. 2013 [115]; Drug Screening Using a Library of Human Induced Pluripotent Stem Cell–Derived Cardiomyocytes Reveals Disease-Specific Patterns of Cardiotoxicity	Troponin T (FACS) Expression of cardiac ion channel: SCN5A, KCND3, CACNA1C, KCNH2, KCNQ1, KCNA5, HCN2, HCN4, KCNJ2, KCNJ3, KCNJ5, KCNJ11, KCNE1, KChIP2 (qRT-PCR)	Verpamil, Alfuzosin, Cisapride Nicorandil	CM/AP assay (compound muscle action potential)	hiPSC-derived cardiomyocytes were shown to model cardiotoxicity more accurately than commercially available hERG cell lines
Nephrotoxicity	Morizane et al. 2015 [129]; Nephron organoids derived from human pluripotent stem cells model kidney development and injury	NPCs: 90% of NPC were positive for SIX2, SALL1, WT1, and PAX2; NPC-derived renal vesicles : 76% were positive for PAX8 and LHX1 segmental markers in nephron—like continuous structures: (A) glomerular podocytes: NPHS1 and PODXL; (B) proximal tubules: LTL and CDH2, (C) loops of Henle/distal tubules: E-Cad/CDH1, UMOD and BRN1	Nephrotoxicants tested on hESCs-derived 3D kidney organoids: Gentamycin (48 h, at 5 mg/mL) Cisplatin (2,6,24 or 48 h; at 5 μM, 50 μM)	KIM-1, LTL, E-Cad (CDH1) (ICC, qRT-PCR)	In gentamicin-treated organoids KIM-1 was expressed at the luminal surface of LTL-positive tubules but was not detected in E-Cad/CDH1-positive cells. qRT-PCR analysis showed gentamycin-caused dose-dependent upregulation of this marker. Cisplatin upregulated KIM-1 expression in LTL-positive cells but also suppressed E-Cad/CDH1 expression, indicating both proximal and distal tubular toxicity
	Kandasamy et al. 2015 [119]; Prediction of drug-induced nephrotoxicity and injury mechanisms with human induced pluripotent stem cell-derived cells and machine learning methods	Proximal tubular-like cells expressed e.g., SIX2, WT1, GDNF, HOXD11, KSP-CAD, AQP1, OAT3, GGT, and other markers expressed along proximal tubular cell development; however some of the main stemness markers were highly also expressed	Nephrotoxicants tested: Aristolochic acid, Arsenic (III) oxide, Bismuth (III) oxide, Cadmium chloride, Cephalosporin C, Cisplatin, Citrinin, Copper (II) chloride, 5-Fluorouracil, Gentamicin, Gold (I) chloride, Lead acetate, Paraquat, Potassium dichromate, Puromycin, Rifampicin, Tacrolimus, Tobramycin; (16 h exposure, at 1, 10, 1000 μg/mL)	IL-6, IL-8 (qRT-PCR, normalized to GAPDH and PPIA)	Nephrotoxicity response in iPSC-derived HPTC-like cells were compared to the corresponding dataset from previous study on cultured human primary HPTC cells Comparative automated unbiased data analysis showed 99.8% and 87.0% training balanced accuracy and test balanced accuracy, respectively
Neurotoxicity	Wheeler et al. 2015 [137]; Modeling Chemotherapeutic Neurotoxicity with Human Induced Pluripotent Stem Cell-Derived Neuronal Cells	Cortical neurons were defined as Tuj1-positive and Nestin-negative (ICC)	4 chemotherapeutics: Cisplatin, Paclitaxel, Vincristine, Hydroxyurea; (72 h exposure, at 0.001–100 μM)	Neurite outgrowth response upon chemotherapeutic treatment	The differences between selected paclitaxel-resistant and paclitaxel-sensitive LCL-derived neurons were significant but only partially correlated with the patient’s initial sensitivity to this chemotherapeutic Reduced TUBB2A sensitized iPSC-derived neurons to paclitaxel

## References

[1] Paul G., Li J.Y., Brundin P. (2002). Stem cells: Hype or hope?. Drug Discov. Today.

[2] Yu J., Thomson J.A. (2008). Pluripotent stem cell lines. Genes Dev..

[3] McNeish J. (2004). Embryonic stem cells in drug discovery. Nat. Rev. Drug Discov..

[4] De Wert G., Mummery C. (2003). Human embryonic stem cells: Research, ethics and policy. Hum. Reprod..

[5] Takahashi K., Yamanaka S. (2006). Induction of pluripotent stem cells from mouse embryonic and adult fibroblast cultures by defined factors. Cell.

[6] Aoi T., Yae K., Nakagawa M., Ichisaka T., Okita K., Takahashi K., Chiba T., Yamanaka S. (2008). Generation of pluripotent stem cells from adult mouse liver and stomach cells. Science.

[7] Takahashi K., Tanabe K., Ohnuki M., Narita M., Ichisaka T., Tomoda K., Yamanaka S. (2007). Induction of pluripotent stem cells from adult human fibroblasts by defined factors. Cell.

[8] Yu J., Vodyanik M.A., Smuga-Otto K., Antosiewicz-Bourget J., Frane J.L., Tian S., Nie J., Jonsdottir G.A., Ruotti V., Stewart R. (2007). Induced pluripotent stem cell lines derived from human somatic cells. Science.

[9] Park I.H., Zhao R., West J.A., Yabuuchi A., Huo H., Ince T.A., Lerou P.H., Lensch M.W., Daley G.Q. (2008). Reprogramming of human somatic cells to pluripotency with defined factors. Nature.

[10] Lowry W.E., Richter L., Yachechko R., Pyle A.D., Tchieu J., Sridharan R., Clark A.T., Plath K. (2008). Generation of human induced pluripotent stem cells from dermal fibroblasts. Proc. Natl. Acad. Sci. USA.

[11] Park I.H., Arora N., Huo H., Maherali N., Ahfeldt T., Shimamura A., Lensch M.W., Cowan C., Hochedlinger K., Daley G.Q. (2008). Disease-specific induced pluripotent stem cells. Cell.

[12] Rao M.S., Malik N. (2012). Assessing iPSC reprogramming methods for their suitability in translational medicine. J. Cell Biochem..

[13] Nakanishi M., Otsu M. (2012). Development of Sendai virus vectors and their potential applications in gene therapy and regenerative medicine. Curr. Gene. Ther..

[14] Ebrahimi B. (2015). Reprogramming barriers and enhancers: Strategies to enhance the efficiency and kinetics of induced pluripotency. Cell Regen..

[15] Lund R.J., Narva E., Lahesmaa R. (2012). Genetic and epigenetic stability of human pluripotent stem cells. Nat. Rev. Genet..

[16] Kyttälä A., Moraghebi R., Valensisi C., Kettunen J., Andrus C., Pasumarthy K.K., Nakanishi M., Nishimura K., Ohtaka M., Weltner J. (2016). Genetic Variability Overrides the Impact of Parental Cell Type and Determines iPSC Differentiation Potential. Stem Cell Rep..

[17] Gage F. (2010). The promise and the challenge of modelling human disease in a dish. EMBO Mol. Med..

[18] Hefti F.F. (2008). Requirements for a lead compound to become a clinical candidate. BMC Neurosci..

[19] Kaitin K.I. (2010). Deconstructing the drug development process: The new face of innovation. Clin. Pharmacol. Ther..

[20] Smietana K., Siatkowski M., Moller M. (2016). Trends in clinical success rates. Nat. Rev. Drug. Discov..

[21] Hay M., Thomas D.W., Craighead J.L., Economides C., Rosenthal J. (2014). Clinical development success rates for investigational drugs. Nat. Biotechnol..

[22] De Vos J., Bouckenheimer J., Sansac C., Lemaître J.M., Assou S. (2016). Human induced pluripotent stem cells: A disruptive innovation. Curr. Res. Transl. Med..

[23] Ellis J., Bhatia M. (2011). iPSC technology: Platform for drug discovery. Clin. Pharmacol. Ther..

[24] Vitale A.M., Wolvetang E., Mackay-Sim A. (2011). Induced pluripotent stem cells: A new technology to study human diseases. Int. J. Biochem. Cell Biol..

[25] Fishburn C.S. (2013). Translational research: The changing landscape of drug discovery. Drug Discov. Today.

[26] Mullane K., Winquist R.J., Williams M. (2014). Translational paradigms in pharmacology and drug discovery. Biochem. Pharmacol..

[27] Philips T., Rothstein J.D., Pouladi M.A. (2014). Preclinical models: Needed in translation? A Pro/Con debate. Mov. Disord..

[28] Wu M., Chen G., Hu B. (2013). Induced pluripotency for translational research. Genom. Proteom. Bioinform..

[29] Zhao X., Modur V., Carayannopoulos L.N., Laterza O.F. (2015). Biomarkers in pharmaceutical research. Clin. Chem..

[30] Strimbu K., Tavel J.A. (2010). What are biomarkers?. Curr. Opin. HIV AIDS.

[31] Cummings J., Ward T.H., Greystoke A., Ranson M., Dive C. (2008). Biomarker method validation in anticancer drug development. Br. J. Pharmacol..

[32] Tateishi K., He J., Taranova O., Liang G., D’Alessio A.C., Zhang Y. (2008). Generation of insulin-secreting islet-like clusters from human skin fibroblasts. J. Biol. Chem..

[33] Song Z., Cai J., Liu Y., Zhao D., Yong J., Duo S., Song X., Guo Y., Zhao Y., Qin H. (2009). Efficient generation of hepatocyte-like cells from human induced pluripotent stem cells. Cell Res..

[34] Zhang J., Wilson G.F., Soerens A.G., Koonce C.H., Yu J., Palecek S.P., Thomson J.A., Kamp T.J. (2009). Functional cardiomyocytes derived from human induced pluripotent stem cells. Circ. Res..

[35] Wong D.F., Potter W.Z., Brasic J.R., Davis K.L., Charney D., Coyle J.T., Nemeroff C. (2002). Proof of concept: Functional models for drug development in humans. Neuropsychopharmacology: The Fifth Generation of Progress.

[36] Wehling M. (2009). Assessing the translatability of drug projects: What needs to be scored to predict success?. Nat. Rev. Drug Discov..

[37] Wendler A., Wehling M. (2012). Translatability scoring in drug development: Eight case studies. J. Transl. Med..

[38] Saha K., Jaenisch R. (2009). Technical challenges in using human induced pluripotent stem cells to model disease. Cell Stem Cell.

[39] Ebert A.D., Yu J., Rose F.F., Mattis V.B., Lorson C.L., Thomson J.A., Svendsen C.N. (2009). Induced pluripotent stem cells from a spinal muscular atrophy patient. Nature.

[40] Lee G., Papapetrou E.P., Kim H., Chambers S.M., Tomishima M.J., Fasano C.A., Ganat Y.M., Menon J., Shimizu F., Viale A. (2009). Modelling pathogenesis and treatment of familial dysautonomia using patient-specific iPSCs. Nature.

[41] Stacey G.N., Crook J.M., Hei D., Ludwig T. (2013). Banking human induced pluripotent stem cells: Lessons learned from embryonic stem cells?. Cell Stem Cell.

[42] Qin Y., Gao W.Q. (2016). Concise review: Patient-derived stem cell research for monogenic disorders. Stem Cells.

[43] Jozefczuk J., Kashofer K., Ummanni R., Henjes F., Rehman S., Geenen S., Wruck W., Regenbrecht C., Daskalaki A., Wierling C. (2012). A systems biology approach to deciphering the etiology of steatosis employing patient-derived dermal fibroblasts and iPS cells. Front. Physiol..

[44] Marchetto M.C., Carromeu C., Acab A., Yu D., Yeo G.W., Mu Y., Chen G., Gage F.H., Muotri A.R. (2010). A model for neural development and treatment of Rett syndrome using human induced pluripotent stem cells. Cell.

[45] Tang X., Kim J., Zhou L., Wengert E., Zhang L., Wu Z., Carromeu C., Muotri A.R., Marchetto M.C., Gage F.H. (2016). KCC2 rescues functional deficits in human neurons derived from patients with Rett syndrome. Proc. Natl. Acad. Sci. USA.

[46] Tanaka T., Takahashi K., Yamane M., Tomida S., Nakamura S., Oshima K., Niwa A., Nishikomori R., Kambe N., Hara H. (2012). Induced pluripotent stem cells from CINCA syndrome patients as a model for dissecting somatic mosaicism and drug discovery. Blood.

[47] Ho R., Sances S., Gowin G., Amoroso M.W., O’Rourke J.G., Sahabian A., Wichterle H., Baloh R.H., Sareen D., Svendsen C.N. (2016). ALS disrupts spinal motor neuron maturation and aging pathways within gene co-expression networks. Nat. Neurosci..

[48] Hockemeyer D., Soldner F., Beard C., Gao Q., Mitalipova M., DeKelver R.C., Katibah G.E., Amora R., Boydston E.A., Zeitler B. (2009). Efficient targeting of expressed and silent genes in human ESCs and iPSCs using zinc-finger nucleases. Nat. Biotechnol..

[49] Liu G.H., Suzuki K., Qu J., Sancho-Martinez I., Yi F., Li M., Kumar S., Nivet E., Kim J., Soligalla R.D. (2011). Targeted gene correction of laminopathy-associated LMNA mutations in patient-specific iPSCs. Cell Stem Cell.

[50] Grobarczyk B., Franco B., Hanon K., Malgrange B. (2015). Generation of isogenic human iPS cell line precisely corrected by genome editing using the CRISPR/Cas9 system. Stem Cell Rev..

[51] Lin M., Hrabovsky A., Pedrosa E., Wang T., Zheng D., Lachman H.M. (2012). Allele-biased expression in differentiating human neurons: Implications for neuropsychiatric disorders. PLoS ONE.

[52] Chae J.I., Kim D.W., Lee N., Jeon Y.J., Jeon I., Kwon J., Kim J., Soh Y., Lee D.S., Seo K.S. (2012). Quantitative proteomic analysis of induced pluripotent stem cells derived from a human Huntington’s disease patient. Biochem. J..

[53] Harris V.K., Sadiq S.A. (2014). Biomarkers of therapeutic response in multiple sclerosis: Current status. Mol. Diagn. Ther..

[54] Bala S., Petrasek J., Mundkur S., Catalano D., Levin I., Ward J., Alao H., Kodys K., Szabo G. (2012). Circulating microRNAs in exosomes indicate hepatocyte injury and inflammation in alcoholic, drug-induced, and inflammatory liver diseases. Hepatology.

[55] Miller J.D., Ganat Y.M., Kishinevsky S., Bowman R.L., Liu B., Tu E.Y., Mandal P.K., Vera E., Shim J.W., Kriks S. (2013). Human iPSC-based modeling of late-onset disease via progerin-induced aging. Cell Stem Cell.

[56] Carrigan P., Krahn T. (2016). Impact of biomarkers on personalized medicine. Handb. Exp. Pharmacol..

[57] Limpitikul W.B., Dick I.E., Tester D., Boczek N.J., Limphong P., Yang W., Choi M.H., Babich J., DiSilvestre D., Kanter R.J. (2016). A Precision Medicine Approach to the Rescue of Function on Malignant Calmodulinopathic Long QT Syndrome. Circ. Res..

[58] Mertens J., Wang Q.W., Kim Y., Yu D.X., Pham S., Yang B., Zheng Y., Diffenderfer K.E., Zhang J., Soltani S. (2015). Differential responses to lithium in hyperexcitable neurons from patients with bipolar disorder. Nature.

[59] Kawasaki Y., Oda H., Ito J., Niwa A., Tanaka T., Hijikata A., Seki R., Nagahashi A., Osawa M., Asaka I. (2016). Pluripotent cell-based phenotypic dissection identifies a high-frequency somatic NLRC4 mutation as a cause of autoinflammation. Arthritis Rheumatol..

[60] Inoue H., Nagata N., Kurokawa H., Yamanaka S. (2014). iPS cells: A game changer for future medicine. EMBO J..

[61] Swinney D.C., Anthony J. (2011). How were new medicines discovered?. Nat. Rev. Drug Discov..

[62] Engel M., Do-Ha D., Muñoz S.S., Ooi L. (2016). Common pitfalls of stem cell differentiation: A guide to improving protocols for neurodegenerative disease models and research. Cell. Mol. Life Sci..

[63] Rashid S.T., Corbineau S., Hannan N., Marciniak S.J., Miranda E., Alexander G., Huang-Doran I., Griffin J., Ahrlund-Richter L., Skepper J. (2010). Modeling inherited metabolic disorders of the liver using human induced pluripotent stem cells. J. Clin. Investig..

[64] Janzen W.P. (2014). Screening technologies for small molecule discovery: The state of the art. Chem. Biol..

[65] Fraietta I., Gasparri F. (2016). The development of high-content screening (HCS) technology and its importance to drug discovery. Expert Opin. Drug Discov..

[66] Tang S., Xie M., Cao N., Ding S. (2016). Patient-specific induced pluripotent stem cells for disease modeling and phenotypic drug discovery. J. Med. Chem..

[67] Muller P.Y., Milton M.N. (2012). The determination and interpretation of the therapeutic index in drug development. Nat. Rev. Drug Discov..

[68] Lee H., Lee J.W. (2016). Target identification for biologically active small molecules using chemical biology approaches. Arch. Pharm. Res..

[69] Wagner B.K., Schreiber S.L. (2016). The power of sophisticated phenotypic screening and modern mechanism-of-action methods. Cell Chem. Biol..

[70] Belda I., Penas G., Alonso A., Marquina D., Navascués E., Santos A. (2014). Biotech patents and science policy: The Spanish experience. Nat. Biotechnol..

[71] Kaufmann M., Schuffenhauer A., Fruh I., Klein J., Thiemeyer A., Rigo P., Gomez-Mancilla B., Heidinger-Millot V., Bouwmeester T., Schopfer U. (2015). High-Throughput Screening Using iPSC-Derived Neuronal Progenitors to Identify Compounds Counteracting Epigenetic Gene Silencing in Fragile X Syndrome. J. Biomol. Screen..

[72] Jacoby E., Tresadern G., Bembenek S., Wroblowski B., Buyck C., Neefs J.M., Rassokhin D., Poncelet A., Hunt J., van Vlijmen H. (2015). Extending kinome coverage by analysis of kinase inhibitor broad profiling data. Drug Discov. Today.

[73] Durham T.B., Blanco M.J. (2015). Target engagement in lead generation. Bioorg. Med. Chem. Lett..

[74] Kikuchi T., Morizane A., Doi D., Onoe H., Hayashi T., Kawasaki T., Saiki H., Miyamoto S., Takahashi J. (2011). Survival of human induced pluripotent stem cell-derived midbrain dopaminergic neurons in the brain of a primate model of Parkinson’s disease. J. Parkinson’s Dis..

[75] Lippert J., Burghaus R., Kuepfer L., Ploeger B., Schaller S., Schmitt W., Willmann S. (2016). Modeling and Simulation of In Vivo Drug Effects. Handb. Exp. Pharmacol..

[76] Iwao T., Toyota M., Miyagawa Y., Okita H., Kiyokawa N., Akutsu H., Umezawa A., Nagata K., Matsunaga T. (2014). Differentiation of human induced pluripotent stem cells into functional enterocyte-like cells using a simple method. Drug Metab. Pharmacokinet..

[77] Minami H., Tashiro K., Okada A., Hirata N., Yamaguchi T., Takayama K., Mizuguchi H., Kawabata K. (2015). Generation of Brain Microvascular Endothelial-Like Cells from Human Induced Pluripotent Stem Cells by Co-Culture with C6 Glioma Cells. PLoS ONE.

[78] Katt M.E., Xu Z.S., Gerecht S., Searson P.C. (2016). Human Brain Microvascular Endothelial Cells Derived from the BC1 iPS Cell Line Exhibit a Blood-Brain Barrier Phenotype. PLoS ONE.

[79] Takasato M., Er P.X., Chiu H.S., Maier B., Baillie G.J., Ferguson C., Parton R.G., Wolvetang E.J., Roost M.S., Chuva de Sousa Lopes S.M., Little M.H. (2015). Kidney organoids from human iPS cells contain multiple lineages and model human nephrogenesis. Nature.

[80] Yamamoto S., Otsu M., Matsuzaka E., Konishi C., Takagi H., Hanada S., Mochizuki S., Nakauchi H., Imai K., Tsuji K. (2015). Screening of drugs to treat 8p11 myeloproliferative syndrome using patient-derived induced pluripotent stem cells with fusion gene CEP110-FGFR1. PLoS ONE.

[81] Aronson J.K. (2005). Biomarkers and surrogate endpoints. Br. J. Clin. Pharmacol..

[82] Luni C., Serena E., Elvassore N. (2014). Human-on-chip for therapy development and fundamental science. Curr. Opin. Biotechnol..

[83] Skardal A., Shupe T., Atala A. (2016). Organoid-on-a-chip and body-on-a-chip systems for drug screening and disease modeling. Drug Discov. Today.

[84] Wendler A., Wehling M. (2010). The translatability of animal models for clinical development: Biomarkers and disease models. Curr. Opin. Pharmacol..

[85] Lee W.M. (2003). Drug-induced hepatotoxicity. N. Engl. J. Med..

[86] Kaplowitz N. (2005). Idiosyncratic drug hepatotoxicity. Nat. Rev. Drug Discov..

[87] Roth R.A., Ganey P.E. (2010). Intrinsic versus idiosyncratic drug-induced hepatotoxicity—Two villains or one?. J. Pharmacol. Exp. Ther..

[88] Gerets H.H., Tilmant K., Gerin B., Chanteux H., Depelchin B.O., Dhalluin S., Atienzar F.A. (2012). Characterization of primary human hepatocytes, HepG2 cells, and HepaRG cells at the mRNA level and CYP activity in response to inducers and their predictivity for the detection of human hepatotoxins. Cell Biol. Toxicol..

[89] Kanebratt K.P., Andersson T.B. (2008). HepaRG cells as an in vitro model for evaluation of cytochrome P450 induction in humans. Drug Metab. Dispos..

[90] Kanebratt K.P., Andersson T.B. (2008). Evaluation of HepaRG cells as an in vitro model for human drug metabolism studies. Drug Metab. Dispos..

[91] Anson B.D., Kolaja K.L., Kamp T.J. (2011). Opportunities for use of human iPS cells in predictive toxicology. Clin. Pharmacol. Ther..

[92] Park H.J., Choi Y.J., Kim J.W., Chun H.S., Im I., Yoon S., Han Y.M., Song C.W., Kim H. (2015). Differences in the Epigenetic Regulation of Cytochrome P450 Genes between Human Embryonic Stem Cell-Derived Hepatocytes and Primary Hepatocytes. PLoS ONE.

[93] Tanimizu N., Nishikawa M., Saito H., Tsujimura T., Miyajima A. (2003). Isolation of hepatoblasts based on the expression of Dlk/Pref-1. J. Cell Sci..

[94] Coleman M.D. (2010). Human Drug Metabolism: An Introduction.

[95] Mann D.A. (2015). Human induced pluripotent stem cell-derived hepatocytes for toxicology testing. Expert Opin. Drug. Metab. Toxicol..

[96] Lu J., Einhorn S., Venkatarangan L., Miller M., Mann D.A., Watkins P.B., LeCluyse E. (2015). Morphological and Functional Characterization and Assessment of iPSC-Derived Hepatocytes for In Vitro Toxicity Testing. Toxicol. Sci..

[97] Tasnim F., Toh Y.C., Qu Y., Li H., Phan D., Narmada B.C., Ananthanarayanan A., Mittal N., Meng R.Q., Yu H. (2016). Functionally Enhanced Human Stem Cell Derived Hepatocytes in Galactosylated Cellulosic Sponges for Hepatotoxicity Testing. Mol. Pharm..

[98] Ware B.R., Berger D.R., Khetani S.R. (2015). Prediction of Drug-Induced Liver Injury in Micropatterned Co-cultures Containing iPSC-Derived Human Hepatocytes. Toxicol. Sci..

[99] Espejel S., Roll G.R., McLaughlin K.J., Lee A.Y., Zhang J.Y., Laird D.J., Okita K., Yamanaka S., Willenbring H. (2010). Induced pluripotent stem cell-derived hepatocytes have the functional and proliferative capabilities needed for liver regeneration in mice. J. Clin. Investig..

[100] Schomaker S., Warner R., Bock J., Johnson K., Potter D., van Winkle J., Aubrecht J. (2013). Assessment of emerging biomarkers of liver injury in human subjects. Toxicol. Sci..

[101] Pirola C.J., Fernández Gianotti T., Castaño G.O., Mallardi P., San Martino J., Ledesma M.M.G.L., Flichman D., Mirshahi F., Sanyal A.J., Sookoian S. (2015). Circulating microRNA signature in non-alcoholic fatty liver disease: From serum non-coding RNAs to liver histology and disease pathogenesis. Gut.

[102] Enache L.S., Enache E.L., Ramière C., Diaz O., Bancu L., Sin A., André P. (2014). Circulating RNA molecules as biomarkers in liver disease. Int. J. Mol. Sci..

[103] Takebe T., Zhang R.R., Koike H., Kimura M., Yoshizawa E., Enomura M., Koike N., Sekine K., Taniguchi H. (2014). Generation of a vascularized and functional human liver from an iPSC-derived organ bud transplant. Nat. Protoc..

[104] Zhang R.R., Takebe T., Miyazaki L., Takayama M., Koike H., Kimura M., Enomura M., Zheng Y.W., Sekine K., Taniguchi H. (2014). Efficient hepatic differentiation of human induced pluripotent stem cells in a three-dimensional microscale culture. Methods Mol. Biol..

[105] Ogawa S., Surapisitchat J., Virtanen C., Ogawa M., Niapour M., Sugamori K.S., Wang S., Tamblyn L., Guillemette C., Hoffmann E. (2013). Three-dimensional culture and cAMP signaling promote the maturation of human pluripotent stem cell-derived hepatocytes. Development.

[106] Takayama K., Kawabata K., Nagamoto Y., Kishimoto K., Tashiro K., Sakurai F., Tachibana M., Kanda K., Hayakawa T., Furue M.K. (2013). 3D spheroid culture of hESC/hiPSC-derived hepatocyte-like cells for drug toxicity testing. Biomaterials.

[107] McCarty W.J., Usta O.B., Yarmush M.L. (2016). A Microfabricated Platform for Generating Physiologically-Relevant Hepatocyte Zonation. Sci. Rep..

[108] Bhatia S.N., Ingber D.E. (2014). Microfluidic organs-on-chips. Nat. Biotechnol..

[109] Vernetti L.A., Senutovitch N., Boltz R., DeBiasio R., Shun T.Y., Gough A., Taylor D.L. (2016). A human liver microphysiology platform for investigating physiology, drug safety, and disease models. Exp. Biol. Med..

[110] Potta S.P., Šarić T., Heke M., Bahudhanapati H., Hescheler J., Atwood C.S., Meethal S.V. (2014). Human Pluripotent Stem Cell Applications in Drug Discovery and Toxicology—An overview. Pluripotent Stem Cell Biology—Advances in Mechanisms, Methods and Models.

[111] Cheng H., Kar G., Dicker A.P., Rodeck U., Koch W.J., Force T. (2011). A novel preclinical strategy for identifying cardiotoxic kinase inhibitors and mechanisms of cardiotoxicity. Circ. Res..

[112] Force T., Kolaja K.L. (2011). Cardiotoxicity of kinase inhibitors: The prediction and translation of preclinical models to clinical outcomes. Nat. Rev. Drug Discov..

[113] Haraguchi Y., Ohtsuki A., Oka T., Shimizu T. (2015). Electrophysiological analysis of mammalian cells expressing hERG using automated 384-well-patch-clamp. BMC Pharmacol. Toxicol..

[114] Chaudhari U., Nemade H., Wagh V., Gaspar J.A., Ellis J.K., Srinivasan S.P., Spitkovski D., Nguemo F., Louisse J., Bremer S. (2016). Identification of genomic biomarkers for anthracycline-induced cardiotoxicity in human iPSC-derived cardiomyocytes: An in vitro repeated exposure toxicity approach for safety assessment. Arch. Toxicol..

[115] Liang P., Lan F., Lee A.S., Gong T., Sanchez-Freire V., Wang Y., Diecke S., Sallam K., Knowles J.W., Wang P.J. (2013). Drug screening using a library of human induced pluripotent stem cell-derived cardiomyocytes reveals disease-specific patterns of cardiotoxicity. Circulation.

[116] Miki K., Endo K., Takahashi S., Funakoshi S., Takei I., Katayama S., Toyoda T., Kotaka M., Takaki T., Umeda M. (2015). Efficient Detection and Purification of Cell Populations Using Synthetic MicroRNA Switches. Cell Stem Cell.

[117] Bonventre J.V., Vaidya V.S., Schmouder R., Feig P., Dieterle F. (2010). Next-generation biomarkers for detecting kidney toxicity. Nat. Biotechnol..

[118] Tiong H.Y., Huang P., Xiong S., Li Y., Vathsala A., Zink D. (2014). Drug-induced nephrotoxicity: Clinical impact and preclinical in vitro models. Mol. Pharm..

[119] Kandasamy K., Chuah J.K., Su R., Huang P., Eng K.G., Xiong S., Li Y., Chia C.S., Loo L.H., Zink D. (2015). Prediction of drug-induced nephrotoxicity and injury mechanisms with human induced pluripotent stem cell-derived cells and machine learning methods. Sci. Rep..

[120] Van Meer L., Moerland M., Cohen A.F., Burggraaf J. (2014). Urinary kidney biomarkers for early detection of nephrotoxicity in clinical drug development. Br. J. Clin. Pharmacol..

[121] Fuchs T.C., Hewitt P. (2011). Biomarkers for drug-induced renal damage and nephrotoxicity—An overview for applied toxicology. AAPS J..

[122] Davies J.A. (2015). Self-organized Kidney Rudiments: Prospects for Better in vitro Nephrotoxicity Assays. Biomark. Insights.

[123] Huang J.X., Kaeslin G., Ranal M.V., Blaskovich M.A., Becker B., Butler M.S., Little M.H., Lash L.H., Cooper M.A. (2015). Evaluation of biomarkers for in vitro prediction of drug-induced nephrotoxicity: Comparison of HK-2, immortalized human proximal tubule epithelial, and primary cultures of human proximal tubular cells. Pharmacol. Res. Perspect..

[124] Huang J.X., Blaskovich M.A., Cooper M.A. (2014). Cell- and biomarker-based assays for predicting nephrotoxicity. Expert Opin. Drug Metab. Toxicol..

[125] Human Renal Cells (Normal & Diseased, Lonza, Basel, Switzerland). http://www.lonza.com/products-services/bio-research/primary-cells/human-cells-and-media/renal-cells-and-media/human-renal-cells.aspx.

[126] Taguchi A., Kaku Y., Ohmori T., Sharmin S., Ogawa M., Sasaki H., Nishinakamura R. (2014). Redefining the in vivo origin of metanephric nephron progenitors enables generation of complex kidney structures from pluripotent stem cells. Cell Stem Cell.

[127] Taguchi A., Nishinakamura R. (2015). Nephron reconstitution from pluripotent stem cells. Kidney Int..

[128] Takasato M., Er P.X., Becroft M., Vanslambrouck J.M., Stanley E.G., Elefanty A.G., Little M.H. (2014). Directing human embryonic stem cell differentiation towards a renal lineage generates a self-organizing kidney. Nat. Cell Biol..

[129] Morizane R., Lam A.Q., Freedman B.S., Kishi S., Valerius M.T., Bonventre J.V. (2015). Nephron organoids derived from human pluripotent stem cells model kidney development and injury. Nat. Biotechnol..

[130] Nieskens T.T., Wilmer M.J. (2016). Kidney-on-a-chip technology for renal proximal tubule tissue reconstruction. Eur. J. Pharmacol..

[131] Wilmer M.J., Ng C.P., Lanz H.L., Vulto P., Suter-Dick L., Masereeuw R. (2016). Kidney-on-a-Chip Technology for Drug-Induced Nephrotoxicity Screening. Trends Biotechnol..

[132] Tukker A.M., de Groot M.W., Wijnolts F.M., Kasteel E.E., Hondebrink L., Westerink R.H. (2016). Is the time right for in vitro neurotoxicity testing using human iPSC-derived neurons?. ALTEX.

[133] Bal-Price A.K., Hogberg H.T., Buzanska L., Lenas P., van Vliet E., Hartung T. (2010). In vitro developmental neurotoxicity (DNT) testing: Relevant models and endpoints. Neurotoxicology.

[134] Bal-Price A.K., Hogberg H.T., Buzanska L., Coecke S. (2010). Relevance of in vitro neurotoxicity testing for regulatory requirements: Challenges to be considered. Neurotoxicol. Teratol..

[135] Aday S., Cecchelli R., Hallier-Vanuxeem D., Dehouck M.P., Ferreira L. (2016). Stem Cell-Based Human Blood-Brain Barrier Models for Drug Discovery and Delivery. Trends Biotechnol..

[136] Zehendner C.M., White R., Hedrich J., Luhmann H.J. (2014). A neurovascular blood-brain barrier in vitro model. Methods Mol. Biol..

[137] Wheeler H.E., Wing C., Delaney S.M., Komatsu M., Dolan M.E. (2015). Modeling chemotherapeutic neurotoxicity with human induced pluripotent stem cell-derived neuronal cells. PLoS ONE.

[138] Kamao H., Mandai M., Okamoto S., Sakai N., Suga A., Sugita S., Kiryu J., Takahashi M. (2014). Characterization of human induced pluripotent stem cell-derived retinal pigment epithelium cell sheets aiming for clinical application. Stem Cell Rep..

[139] Sayed N., Liu C., Wu J.C. (2016). Translation of Human-Induced Pluripotent Stem Cells: From Clinical Trial in a Dish to Precision Medicine. J. Am. Coll. Cardiol..

[140] Banerjee A., Chaudhury S. (2010). Statistics without tears: Populations and samples. Ind. Psychiatry J..

[141] Kunisada Y., Tsubooka-Yamazoe N., Shoji M., Hosoya M. (2012). Small molecules induce efficient differentiation into insulin-producing cells from human induced pluripotent stem cells. Stem Cell Res..

[142] Baden M.Y., Fukui K., Hosokawa Y., Iwahashi H., Imagawa A., Shimomura I. (2015). Examination of a Viral Infection Mimetic Model in Human iPS Cell-Derived Insulin-Producing Cells and the Anti-Apoptotic Effect of GLP-1 Analogue. PLoS ONE.

